# Diabetic impact on the neuroaxis: from peripheral neuropathy to central neurodegeneration

**DOI:** 10.3389/fendo.2026.1869759

**Published:** 2026-06-10

**Authors:** Tina Okdahl, Christina Brock

**Affiliations:** 1 Mech-Sense, Department of Gastroenterology and Hepatology, Aalborg University Hospital, Gistrup, Denmark; 2 Department of Clinical Medicine, Aalborg University, Aalborg, Denmark; 3 Steno Diabetes Center North Denmark, Aalborg University Hospital, Gistrup, Denmark

**Keywords:** cardiovascular autonomic neuropathy, central neuropathy, diabetes, diabetic neuropathy, distal symmetric polyneuropathy

## Abstract

**Background:**

Diabetic neuropathy has typically been viewed as a peripheral nerve disorder, most commonly presenting as distal symmetrical polyneuropathy (DSPN). However, accumulating evidence suggests that diabetes affects not only peripheral somatic and autonomic fibers but also the central nervous system, indicating more widespread neurodegenerative processes.

**Aim:**

This narrative review aims to synthesize current knowledge on how diabetes affects the nervous system across the neuroaxis, integrating peripheral, autonomic, and central mechanisms, and to provide an overview of clinical manifestations, diagnostic approaches, and management strategies.

**Results:**

Chronic hyperglycemia induces a range of metabolic and vascular disturbances, including oxidative stress, inflammation, and microvascular dysfunction, which contribute to peripheral nerve injury. These changes affect both small and large fibers, leading to sensory loss, neuropathic pain, and motor impairment. Autonomic involvement is common and manifests as cardiovascular, gastrointestinal, sudomotor, urogenital, and ocular dysfunction. Importantly, diabetes-related neural injury extends beyond the peripheral nervous system. Structural and functional alterations have been demonstrated in the spinal cord, brainstem and brain, including changes in white matter integrity, cortical organization, and functional connectivity. Peripheral and central mechanisms interact bidirectionally, contributing to altered sensory processing and pain modulation.

**Conclusion:**

Diabetic neuropathy should be understood as a disorder of the entire neuroaxis. Integrating peripheral and central aspects is essential to gain a holistic view of diabetic neuropathy and to support the development of more targeted diagnostic and therapeutic strategies.

## 1 Introduction

**Figure 1:**
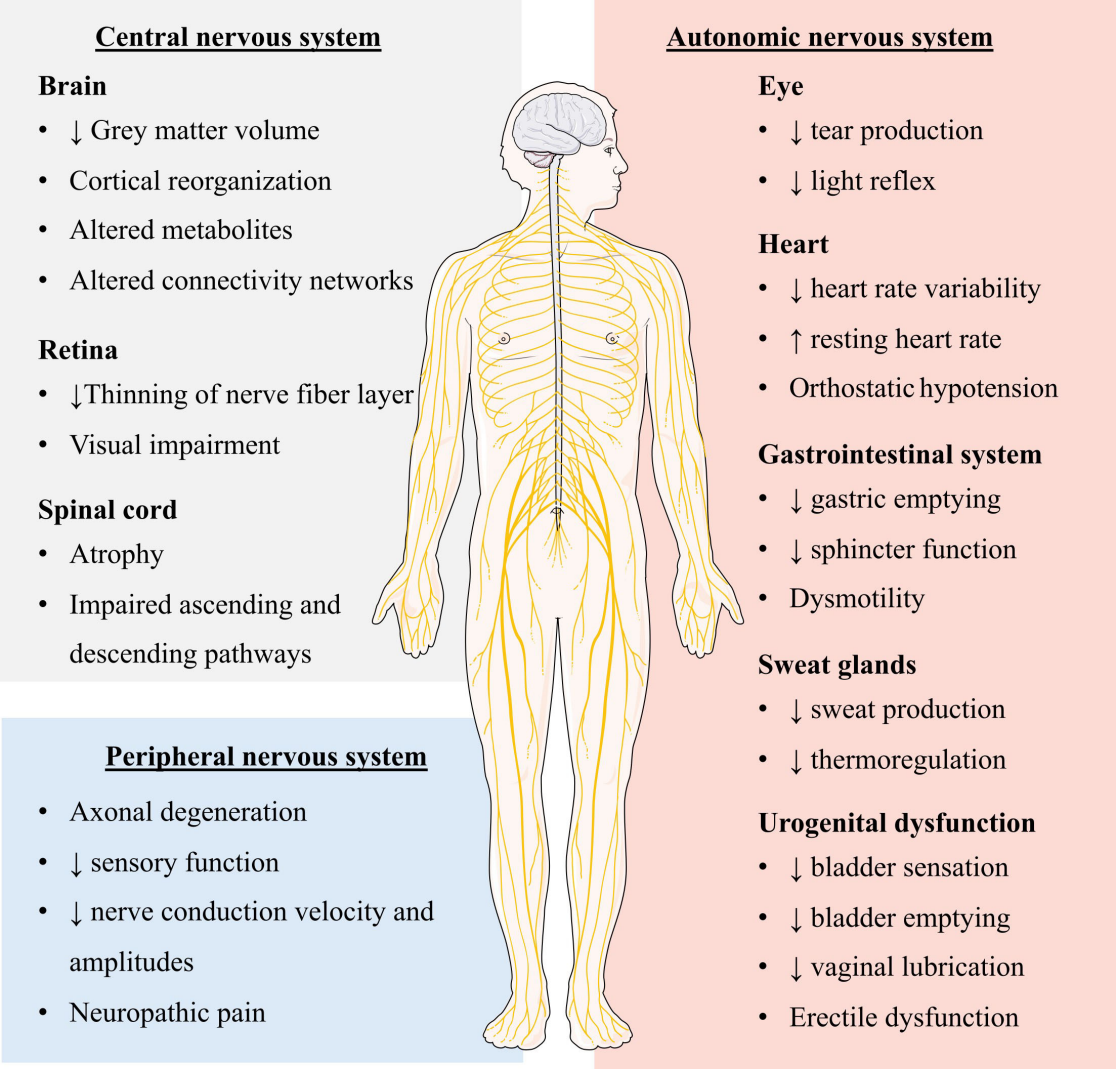
Effects of diabetes on the central (gray), autonomic (red), and peripheral (blue) nervous system.

The global rise in diabetes prevalence, affecting both Western and Eastern populations, has brought long-term complications into sharper clinical and research focus (1, 2). Among these, diabetic neuropathy represents a major burden, both in terms of patient morbidity, self-reported quality of life and healthcare expenditure (3). Traditionally, diabetic neuropathy has been viewed as a disorder primarily affecting the peripheral somatic nerves, most commonly presenting as distal symmetrical polyneuropathy (DSPN). This perspective is largely driven by the high prevalence of DSPN, affecting up to 20% or 50% of individuals with type 1 or type 2 diabetes (4), and by its characteristic clinical features (5). However, this classical view is increasingly recognized as incomplete. Diabetic neuropathy is not confined to the somatic peripheral nervous system but also involves autonomic fibers, and the central nervous system, reflecting a broader neurodegenerative process affecting multiple components of the neuroaxis (6, 7). Accordingly, diabetic neuropathies comprise a heterogeneous group of disorders characterized by impaired nerve function, with clinical manifestations depending on the anatomical distribution and types of nerve fibers involved (**Figure 1**).

Importantly, peripheral nerve damage interacts dynamically with central nervous system function. Reduced or aberrant afferent input from damaged peripheral fibers may drive neuroplastic changes. At the same time, sustained nociceptive signaling and impaired inhibitory control may promote central sensitization, contributing to the persistence and amplification of symptoms such as neuropathic pain (8–10). Moreover, direct effects of chronic hyperglycemia on the central nervous system, may independently contribute to neuronal injury within the brain and spinal cord (11, 12). These mechanisms suggest that central and peripheral neurodegeneration may occur in parallel, rather than sequentially, further blurring the distinction between peripheral and central pathology and supporting the concept of diabetes as a disorder affecting the entire neuroaxis.

In this narrative review, we aim to synthesize current knowledge on the effects of diabetes across the neuroaxis ranging from peripheral sensory detection and signal transmission to autonomic involvement, and central nervous system impairment. Furthermore, we provide an overview of current diagnostic approaches and management strategies for diabetic neuropathies.

## 2 Peripheral neuropathy in diabetes

The peripheral nervous system is comprised of somatic and autonomic branches. The somatic nervous system mediates sensory perception and voluntary motor control, whereas the autonomic nervous system regulates involuntary functions of internal organs, including cardiovascular and gastrointestinal activity (13). Sensory transduction begins at the specialized nerve endings, where distinct receptor types respond to mechanical, thermal, and chemical stimuli and convert these signals into electrical activity that is transmitted centrally (14).

Peripheral nerves are especially susceptible to metabolic dysregulation, as neurons have high and continuous energy demands and rely heavily on glucose metabolism for ATP production. In diabetes, chronic hyperglycemia leads to excessive intracellular glucose availability, overwhelming normal metabolic pathways and initiating a cascade of maladaptive processes that collectively contribute to neuronal injury (15). Increased activity through the polyol pathway leads to intracellular accumulation of sorbitol and depletion of NADPH, disrupting osmotic balance and reducing antioxidant capacity (16, 17). In parallel, enhanced formation of advanced glycation end products (AGEs) results in structural modification of proteins and lipids and activation of pro-inflammatory signaling via AGE receptors (18). Additional pathways, including activation of the hexosamine pathway and protein kinase C, further contribute to altered gene expression, vascular dysfunction, and inflammation (16, 19). Taken together, these metabolic disturbances cause a state of oxidative stress, driven by both mitochondrial dysfunction and impaired antioxidant defenses. Excess substrate delivery to the mitochondria increases production of reactive oxygen species, leading to damage of cellular components and impaired bioenergetics. Reduced ATP availability, in turn, compromises axonal transport and neuronal maintenance, promoting axonal degeneration (20, 21). These alterations may preferentially affect small nociceptive fibers, contributing to abnormal sensory processing, including spontaneous pain, numbness, allodynia, and hyperalgesia.

In addition to direct neuronal injury, diabetes affects the microvasculature supplying peripheral nerves. Endothelial dysfunction, reduced nitric oxide bioavailability, and structural vascular changes impair endoneurial blood flow, resulting in chronic ischemia and hypoxia (22). This further exacerbates metabolic stress within the nerve and contributes to both small and large fiber loss. Moreover, diabetes also alters the functional properties of peripheral nerve signaling. Impaired axonal transport disrupts the delivery of essential proteins and organelles required for neuronal maintenance and signal transmission. Structural changes such as demyelination and axonal degeneration further impair conduction velocity, particularly in large myelinated fibers (23). At the same time, injured sensory fibers may develop abnormal excitability, including spontaneous ectopic activity and altered ion channel expression (7). Furthermore, alterations in neurotransmitter release and synaptic function at the level of primary afferent terminals may modify signal transmission before it reaches the spinal cord (24). These combined alterations affect signal transmission from peripheral receptors via dorsal root ganglia to second-order neurons in the spinal dorsal horn, and collectively, these changes result in both reduced and distorted afferent input.

## 3 Central neurodegeneration in diabetes

### 3.1 Spinal cord

The spinal cord represents a key relay in the transmission and modulation of somatosensory information. In diabetes, structural alterations of the spinal cord have been reported, including reduced cross-sectional area suggestive of atrophy, as well as changes consistent with demyelination and axonal degeneration (25). Functionally, both ascending and descending pathways appear to be affected. Impairment of ascending spinothalamic and dorsal column pathways may contribute to altered sensory processing, including reduced detection thresholds and impaired discrimination (25, 26). At the same time, disruption of descending inhibitory pathways originating from supraspinal centers may reduce endogenous pain modulation (27). This imbalance between diminished afferent input and impaired central inhibition may contribute to the paradoxical coexistence of sensory loss and neuropathic pain observed in diabetic neuropathy.

### 3.2 Brain

At the supraspinal level, neuroimaging studies have demonstrated widespread structural and functional alterations in individuals with diabetes. Reductions in gray matter volume have been reported in several regions involved in sensory processing and pain modulation (28, 29). These changes may reflect both neurodegenerative processes and adaptive or maladaptive cortical reorganization in response to altered peripheral input. Also, cortical reorganization has been observed in both primary and secondary somatosensory areas, where altered representations of the body may arise following chronic changes in afferent signaling (30). In addition, changes in thalamic volume have been associated with increased pain severity (31), highlighting the role of this key relay structure in modulating sensory input and pain perception.

Microvascular alterations also affect the brain and may contribute to neuronal injury and dysfunction. Cerebral small vessel disease, reduced perfusion, and blood–brain barrier disruption have all been implicated in the pathophysiology of central involvement in diabetes (32, 33). Complementing these structural findings, magnetic resonance spectroscopy studies have demonstrated alterations in brain metabolites, including markers of neuronal integrity and glial activity, suggesting ongoing neurochemical changes (34).

Diffusion tensor imaging has provided further insight into white matter integrity, revealing altered microstructural properties along major sensory pathways (35). Changes in diffusion metrics, such as fractional anisotropy and mean diffusivity, indicate impairments of axonal organization and myelination (36). Importantly, these alterations often follow trajectories consistent with known neuroanatomical pathways, supporting the concept of a continuous neurodegenerative process extending from the periphery to central structures.

Beyond structural alterations, diabetes is associated with changes in central processing and network-level organization. Functional neuroimaging studies have demonstrated altered connectivity within and between brain networks involved in sensory processing, salience detection, and pain modulation (37, 38). These changes may reflect both compensatory mechanisms and maladaptive plasticity. Central sensitization, characterized by increased responsiveness of central neurons to peripheral input, has also been proposed as a mechanism contributing to neuropathic pain in diabetes (10). However, also impairments in cortical response to ascending sensory inputs have been shown (39). Importantly, central and peripheral mechanisms are closely intertwined (40). Altered input from damaged peripheral nerves can drive central reorganization, while central dysfunction may, in turn, influence peripheral sensitivity and symptom perception (8). This bidirectional interaction underscores the importance of considering diabetic neuropathy as a disorder of the entire neuroaxis rather than confined to isolated anatomical compartments.

### 3.3 Retina

The retina represents a unique extension of the central nervous system and offers a non-invasive view of neurodegenerative processes in diabetes. Beyond the well-characterized microvascular changes seen in diabetic retinopathy (41), there is increasing evidence of retinal neurodegeneration with thinning of the retinal nerve fiber layer, reflecting loss of retinal ganglion cell axons (42). Importantly, these neural changes may occur early in the disease course and have been reported even in individuals without overt retinopathy, suggesting that neurodegeneration may precede or occur independently of microvascular pathology (43). As such, retinal measures may serve as accessible biomarkers of central neurodegeneration (44).

## 4 Manifestations of diabetic neuropathy

### 4.1 Peripheral somatic manifestations

#### 4.1.1 Distal symmetrical polyneuropathy

DSPN is a length-dependent neuropathy that typically begins in the lower extremities and progresses proximally. Clinically, DSPN is associated with sensory loss, allodynia, neuropathic pain, and an increased risk of foot ulceration and amputations (5). Early manifestations often include paresthesia, numbness, and impaired temperature or pain perception, reflecting primarily involvement of small sensory fibers (45). In parallel, involvement of large myelinated fibers may lead to impaired vibration sense, proprioceptive deficits, and reduced or absent deep tendon reflexes and ultimately the large motor fibers may also be involved leading to muscle weakness, impaired balance and gait instability (2, 46). This combination of sensory loss and motor involvement contributes substantially to morbidity and risk of falls (47). In up to 30% of people with DSPN the condition is accompanied by neuropathic pain presenting as burning pain, allodynia, or hyperalgesia, reflecting both peripheral sensitization and altered central processing (5, 10, 48).

Distinction between small fiber and large fiber neuropathy is important, as these affect different nerve types and require different diagnostic approaches. Small fiber neuropathy primarily involves thinly myelinated Aδ fibers and unmyelinated C fibers responsible for pain and temperature detection (14), with pain and thermal perception thresholds used for subjective assessment of their integrity (2). For objective assessment, intraepidermal nerve fiber density can be used in which skin biopsies are studied for morphological abnormalities such as axonal swellings (49). Moreover, corneal confocal microscopy (CCM) has emerged as a non-invasive modality for screening of small fiber integrity (50). In contrast, large fiber neuropathy affects myelinated Aβ fibers and is characterized by impaired conduction velocity, vibration sense, proprioception, and reduced reflexes (14). Evaluation typically includes clinical examination of light touch and vibration perception, assessment of deep tendon reflexes, and nerve conduction studies measuring conduction velocity and amplitude (2). The clinical diagnosis of DSPN relies on a combination of characteristic subjective symptoms and objective signs, as summarized in the Toronto Consensus criteria (5). When screening for DSPN, vitamin B12 deficiency should always be considered as a differential diagnosis, because it may have clinical manifestations resembling peripheral, autonomic, and even painful neuropathy (51). Moreover, serum vitamin B12 alone may underestimate functional deficiency, whereas methylmalonic acid and homocysteine may provide greater diagnostic sensitivity (52).

Optimization of glycemic control remains a cornerstone in preventing and stalling the progression of DSPN, although neuropathy may develop despite good glycemic control, particularly in type 2 diabetes (53). Lifestyle interventions, including physical activity and weight management, may have beneficial effects on metabolic and vascular risk factors (4). Painful DSPN is managed with pharmacological therapies targeting neuropathic pain, such as duloxetine, pregabalin, gabapentin and tricyclic antidepressants, as monotherapies or in combination (54) depending on patient profile and tolerability (5). However, a significant proportion of affected individuals are refractory to the available pain management. For these patients, neuromodulation may constitute an novel alternative (55). Disease-modifying management for DSPN with or without pain remains clinically unavailable, but a high number of clinical trials are currently investigating therapeutics targeting underlying mechanisms such as inflammation, oxidative stress, and dyslipidemia (56).

### 4.2 Autonomic manifestations

#### 4.2.1 Cardiovascular autonomic neuropathy

Cardiovascular autonomic neuropathy (CAN) is characterized by impaired autonomic neuro-regulation of heart rate and vascular tone, primarily due to dysfunction of vagal and sympathetic pathways. This results in reduced heart rate variability and a loss of physiological adaptability to internal and external demands, such as changes in posture or physical activity. Eventually, the heart rhythm becomes chronometrically, and in advanced stages, this may contribute to exercise intolerance, orthostatic hypotension, and increased cardiovascular risk (57, 58). In severe cases, CAN is associated with an increased risk of silent myocardial ischemia and cardiac arrhythmias (59). Importantly, CAN has also been associated with left ventricular diastolic dysfunction and hypertrophy of the left ventricle suggesting that autonomic imbalance is directly linked to diabetic cardiomyopathy (60, 61).

The diagnosis of CAN is based on cardiovascular reflex tests, which assess heart rate and blood pressure responses to standardized physiological challenges (58). Heart rate variability (HRV) analysis can also be used as assessment of CAN. Reduced HRV, particularly decreased time-domain measures such as SDNN and RMSSD, reflects impaired parasympathetic modulation and is an early marker of CAN (5, 62). Symptom assessment may be supported by validated questionnaires such as the Composite Autonomic Symptom Score (COMPASS-31), which captures autonomic dysfunction across multiple domains (63).

Management of CAN focuses on cardiovascular risk reduction and symptom control. This includes optimization of glycemic control, blood pressure, and lipid levels, as well as lifestyle interventions (58).

#### 4.2.2 Gastroenteropathy

The gastrointestinal tract is extensively innervated by the enteric nervous system embedded within the gut wall, as well as by extrinsic sympathetic and parasympathetic pathways. In diabetes, structural disruption of this complex intertwined neural network gives rise to a broad spectrum of symptoms collectively referred to as diabetic gastroenteropathy (64), where chronic hyperglycemia, oxidative stress, and microvascular dysfunction contribute to degeneration of enteric neurons, interstitial cells of Cajal, and vagal pathways (64, 65). While gastroparesis, defined as delayed gastric emptying in the absence of mechanical obstruction (66), is the most widely recognized manifestation (67), involvement of the lower gastrointestinal tract is also common. Impaired secretion of digestive hormones and enzymes and dysmotility in the small and large intestine may lead to disordered peristalsis, reduced sphincter function, and altered intestinal transit (64, 68, 69), resulting in symptoms such as abdominal pain, bloating, diarrhea, constipation, and, in some cases, fecal incontinence all of which may significantly reduce quality of life (70).

The diagnosis of gastroparesis relies on the demonstration of delayed gastric emptying using objective testing, most commonly gastric emptying scintigraphy, which is considered the golden standard (71). Alternative methods include breath tests using stable isotopes and wireless motility capsules, which allow assessment of transit times throughout the gastrointestinal tract (64). Given the involvement of the entire gastrointestinal tract, comprehensive evaluation can also be relevant with pan-alimentary magnetic resonance imaging providing a non-invasive and thorough evaluation of both structural and functional parameters (72). Patient-reported outcome measures (PROMs) play an important complementary role in the evaluation of symptom burden. The Gastroparesis Cardinal Symptom Index is a validated questionnaire that assesses key symptoms (73). While symptom severity does not always correlate closely with objective measures of gastric emptying (69), PROMs are valuable for clinical assessment and for monitoring treatment response over time.

Management of diabetic gastroenteropathy includes dietary modifications, optimization of glycemic control, and pharmacological therapies targeting gastrointestinal motility and symptom relief. Also prokinetic agents and antiemetics may be used (74). In refractory cases, gastric electrical stimulation may be considered (75). An important aspect to consider is the fact that GLP-1 receptor agonists delay gastric emptying and thus may contribute to or exacerbate symptoms of gastroparesis (76). In patients with clinically significant gastroparesis symptoms, reassessment of GLP-1 receptor agonist therapy, including potential dose reduction or discontinuation, may therefore be warranted.

#### 4.2.3 Sudomotor dysfunction

Sudomotor dysfunction is a common manifestation of diabetic autonomic neuropathy, reflecting impaired sympathetic cholinergic innervation of sweat glands in the skin. Clinically, this typically presents as reduced or absent sweating in a length-dependent pattern, most prominently affecting the distal extremities (77, 78). Sudomotor dysfunction may contribute to impaired thermoregulation and increased risk of skin dryness, fissures, and ulceration (79). Sudomotor function can be assessed using quantitative sudomotor axon reflex testing, which measures sweat production in response to acetylcholine-mediated stimulation. Additional methods include electrochemical skin conductance (e.g., Sudoscan) and thermoregulatory sweat testing (78).

#### 4.2.4 Urogenital dysfunction

Urogenital dysfunction in diabetes reflects autonomic involvement of both sympathetic and parasympathetic pathways controlling bladder and sexual function. Diabetic cystopathy is characterized by impaired bladder sensation, reduced detrusor contractility, and incomplete bladder emptying, which may progress to overflow incontinence (80). Sexual dysfunction is also common, including erectile dysfunction in men and reduced vaginal lubrication and dyspareunia in women (81). Urogenital manifestations often develop slowly and may significantly impact quality of life (82, 83).

#### 4.2.5 Ocular autonomic dysfunction

Ocular autonomic dysfunction in diabetes reflects impaired parasympathetic and sympathetic innervation of lacrimal glands and pupillary control. Reduced lacrimal gland stimulation leads to decreased tear production and contributes to dry eye disease (84), while pupillary dysfunction may result in impaired light reflexes and reduced dynamic pupillary responses (85). These changes may coexist with diabetic retinopathy and corneal nerve fiber loss, reflecting combined vascular, somatic, and autonomic involvement of the diabetic eye. Evaluation of ocular autonomic dysfunction includes clinical assessment of tear film stability and pupillometry (84, 85). Sympathetic innervation of tarsal muscles may also be affected and thus measurement of palpebral fissure heights may serve as an indicator of autonomic dysfunction (86). Corneal confocal microscopy may provide additional insight into associated small fiber neuropathy (87).

### 4.3 Central manifestations

#### 4.3.1 Cognitive impairment

Cognitive impairment is increasingly recognized as a central manifestation of diabetes (88, 89). These changes are often subtle in early stages but may progress over time and, in some individuals, contribute to an increased risk of mild cognitive impairment and dementia (90). The underlying mechanisms are multifactorial and include chronic hyperglycemia, microvascular dysfunction, insulin resistance, and neuroinflammation, which together may lead to structural and functional alterations within the brain (91).

#### 4.3.2 Visual impairment

Visual impairment in diabetes extends beyond classical diabetic retinopathy and may also reflect neurodegenerative changes within the visual system. Whereas retinopathy is primarily a microvascular disorder affecting retinal vessels (41), diabetes-related visual dysfunction may additionally involve neuronal loss and dysfunction within retinal and central visual pathways, contributing to deficits in contrast sensitivity, visual processing, and dark adaptation. Assessment of structural retinal changes is primarily performed using optical coherence tomography (OCT), which allows high-resolution visualization of retinal layers (42). Thinning of the retinal nerve fiber layer and ganglion cell layer may serve as indicators of neurodegenerative involvement, while OCT angiography can provide complementary information on microvascular integrity (92).

## 5 Conclusion

Diabetic neuropathy is a systemic manifestation reflecting both peripheral and central alterations and, as such, should be seen as a condition affecting all levels of the neuroaxis. Peripheral nerve injury, autonomic dysfunction, and central nervous system alterations may occur in parallel and interact through dynamic, bidirectional mechanisms. This integrated perspective is supported by advances in neuroimaging, neurophysiology, and clinical phenotyping, demonstrating structural and functional changes from peripheral fibers to spinal and supraspinal pathways. Recognizing this complexity is essential for improving diagnosis and treatment. Future research should therefore integrate peripheral and central aspects to gain a holistic view of diabetic neuropathy and enable more targeted, mechanism-based interventions.
